# Automated Microfluidic Blood Lysis Protocol for Enrichment of Circulating Nucleated Cells

**DOI:** 10.3791/1656

**Published:** 2009-12-31

**Authors:** William N. White, Palaniappan Sethu

**Affiliations:** Department of Mechanical Engineering, University of Louisville; Department of Bioengineering, University of Louisville

## Abstract

In this report the protocol for an automated microfluidic blood lysis device is detailed. Circulating nucleated cells (CNCs), including leukocytes and endothelial cells, provide an ideal platform for an updated status on the immune condition of an individual. The microfluidic protocol allows for enrichment of CNCs without selective cell loss and sample preparation variability due to user-mediated steps. Briefly, the protocol includes device fabrication, sample collection, device setup, and running blood through the microfluidic chamber. Within the device whole blood is rapidly mixed with deionized water for approximately 10 seconds in a 50 micron x 150 micron microfluidic channel. In this time span erythrocytes are lysed due to hypotonic conditions. Herringbone structures on the bottom of the channel ensure thorough mixing and exposure of cells to a constant environment. Remaining cells are returned to isotonic conditions at the exit of the device, fixed using 2% paraformaldehyde, centrifuged to separate erythrocyte debris from CNCs, and suspended in flow buffer for staining and analysis by flow cytometry. Results show clean flow cytometry scatter plots with CNC populations saved. Significance of this device and protocol comes in the study and understanding of disease pathogenesis by analysis of CNC populations. Hence, automation, effectiveness, and simplicity of the microfluidic protocol are demonstrated.

**Figure:**
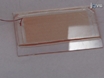


## Protocol

### Part 1. Microfluidic device fabrication

A silicon master mold of the microfluidic lysis device was created using standard photolithographic techniques with the equipment in the University of Louisville cleanroom [1]. AutoCAD (Autodesk, Inc., San Rafael, CA) was used to generate a transparency mask (Fineline Imaging, Colorado Springs, CO) to create negative replicas of the channels. The microfluidic lysis device was fabricated from the silicon master mold using soft lithographic techniques with the elastomer poly(dimethylsiloxane) (PDMS; Dow Corning, Midland, MI). The elastomer with the replicated channels was released, and channel access holes were punched with a 20-gauge needle (Small Parts, Miramar, FL.). The PDMS wafer was irreversibly bonded to a glass slide via oxygen plasma and tested for experimentation. 

### Part 2. Protocol for running lysis device

After the microfluidic device has been fabricated and tested, experiments with erythrocyte lysis are ready to begin. The following section details the protocol for the microfluidic device.

#### 2.1 Sample Collection

Collect 4 milliliters of blood sample from median cubital vein, on the anterior forearm vein of patient with heparin as anticoagulant in two 2 ml green top vaccutainers.
Discard first 2 ml tube as it contains dislodged mature endothelial cells (false positives).
Resuspend the second tube to mix heparin with blood by turning up and down; save immediately on ice.
Transfer 1 ml of blood to a 1.5 ml Eppendorf tube. Process the blood sample immediately (within one hour of collection).

#### 2.2 Prime microfluidic device with PBS

Obtain a microfluidic device bonded to glass. Press-fit access tubing (Small Parts, Miramar, FL) of slightly larger diameter in the inlets and outlet (Figure 1).
Attach a 30-gauge syringe needle (Small Parts, Miramar, FL) to the tubing on each of the inlets, providing the macro to micro interface.
Fill a 1 ml syringe with 1X PBS and make sure to get rid of bubbles by flicking the syringe needle.  Connect the PBS-loaded 1 ml syringe to inlet 1 on the microfluidic device.  Push the syringe containing the 1X  PBS gently by hand until the solution flows out of inlet 2, and then immediately clamp inlet 2 with a standard office binder clip
Continue pushing the 1X PBS until fluid reaches the outlet port of the microfluidics device. Once the solution flows out of the outlet, clamp the outlet tubing with another binder clip.
Continue pushing the 1 ml syringe until the 1X PBS flows out of inlet 3, and clamp the inlet 3 port with another office binder clip.  The microfluidics device is now fully primed.

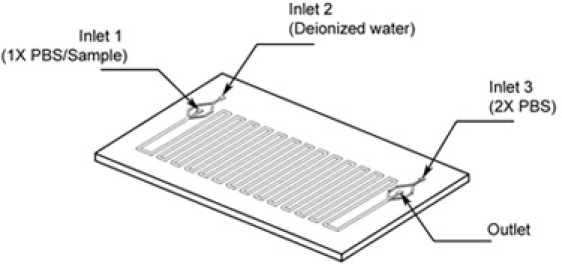
 **Figure 1.** Schematic of device showing inlets for respective solutions and outlet.

#### 2.3 Preparation of syringe pumps and solution

To calibrate the pumps follow these steps:
  For the large Harvard syringe pump (Harvard, PHD 22/2000 Part # 702000): Set the diameter configuration to 22.5 mm, flow rate 600 μl/min.
  For the small Harvard Syringe pump (Harvard, Pico Plus Part # 702213): Set the diameter configuration to 4.61 mm, flow rate 20 μl/min.
Fill one 30 ml syringe with sterile deionized water. This can be accomplished by withdrawing the solution directly from the 50 ml conical tube. (Label the syringe)
Fill a second 30 ml syringe with 2X phosphate-buffered-saline (PBS), 2% paraformaldehyde (PFA) using the procedure outlined in step 9. (Label the syringe)
Fill a 1 ml syringe with 1X PBS from the aliquots stored in the 15 ml conical tubes, using the procedure outlined in step 9.
Connect the 30 ml deionized water syringe to inlet 2 and the 30 ml 2X PBS syringe to inlet 3 (make sure you don t trap bubbles in the tubing or microfluidics device). Remove the clamp from inlets 2 and 3 and the outlet tubing. Connect the 1X PBS 1 ml syringe into inlet 1. Avoid introducing bubbles by filling the syringe needle with liquid, connecting the syringe, and flicking the syringe needle to rid of bubbles.

#### 2.4 Erythrocyte lysis

Set all of the syringes on the proper Harvard pumps as outlined below:
  Set the two 30 ml syringes (one containing sterile, de-ionized water and the other with 2X PBS, 2% PFA) together on the large Harvard syringe pump.
  Set the 1 ml syringe (containing 1X PBS) on the small Harvard syringe pump.
  Place the outlet tubing in a waste collector (50 ml conical tube that is labeled  waste ).
Turn on the large Harvard syringe pump (with the 30 ml syringes) and let it run for one minute. (Make sure there are no bubbles and no leakage in the device or in the tubing).
Turn on the small Harvard syringe pump and let it run for an additional minute. (Make sure there are no bubbles and no leakage in the device or in the tubing). Stop the pumps. The microfluidics device is now ready to be used.
Remove the 1 ml syringe containing 1X PBS from the small Harvard syringe pump.
After obtaining a blood sample, fill the 1 ml syringe with 0.05 ml of sterile 1X PBS without trapping any bubbles. Next, fill the syringe with 0.5 ml of blood obtained from the Eppendorf tube. (The 1 ml syringe will now contain a final volume of 0.55 ml of the blood and 1X PBS buffer.)  Note: Keep the syringe vertical while filling to avoid mixing of the 0.05 ml PBS with the blood.
Connect the syringe containing the blood to inlet 1 and mount the syringe carefully (avoid pushing the blood through the tubing into the device) on the small Harvard syringe pump (the syringe is mounted vertical into the pump).
Remove the outlet tubing from the  waste  tube and put a clean 50 ml centrifuge tube in its place and set it on a bucket of ice.
Switch on the large Harvard syringe pump first and let it run for 1 minute. Start collecting the sample from the outlet into the 50 ml centrifuge tube.
Switch on the small Harvard syringe pump.
Once the blood sample in the 1 ml syringe has completely traveled through the device, stop both pumps.  This should take approximately 20 minutes.
Centrifuge the collected sample for 5 minutes at 350 x g at room temperature with the brake off.
Remove supernatant by placing pipette tip at the opposite side of the white pellet. Remove as much supernatant as possible, especially red cell debris, without disturbing the white pellet.
Resuspend the sample in 1 ml of flow buffer (1X PBS, 1% BSA [bovine serum albumin], 2% PFA) by pipetting up and down. Transfer sample to a 1.5 ml Eppendorf tube.
Microcentrifuge the collected sample for 5 minutes at 4 x g at room temperature. Use same precision as before to remove the supernatant.
Resuspend the sample in 1 ml of flow buffer by vortexing.

#### 2.5 Preparation for flow cytometric analysis

For sample analysis using flow cytometry, add 100 μl of sample to a flow cytometry tube.
To the 100 μl of sample add specified antibody (ensure correct concentration).
Allow sample to incubate for 30 minutes at 40 C.
Wash twice with flow buffer prior to flow cytometry. Wash step includes adding 250 μl of flow buffer, vortexing, centrifuging for 5 minutes at 350 x g, and removing supernatant with one fluid turn of flow cytometry tube.
Resuspend the pellet in 250 μl of flow buffer. Sample is ready for analysis by flow cytometry.

### Representative Results

Whole blood has been run through the microfluidic lysis device and post-processing protocol, ridding of erythrocytes and enriching circulating nucleated cells (CNCs). Ideal results will yield a clean flow cytometry scatter plot with separate CNC populations. Displayed below in Figures 2 and 3 are scatter plot comparisons of a whole blood sample without erythrocyte lysis and with erythrocyte lysis by the microfluidic protocol with axes as forward scatter (FSC) versus side scatter (SSC) light. It can be seen that the microfluidic enrichment protocol is necessary to obtain a clean flow cytometry scatter plot. 


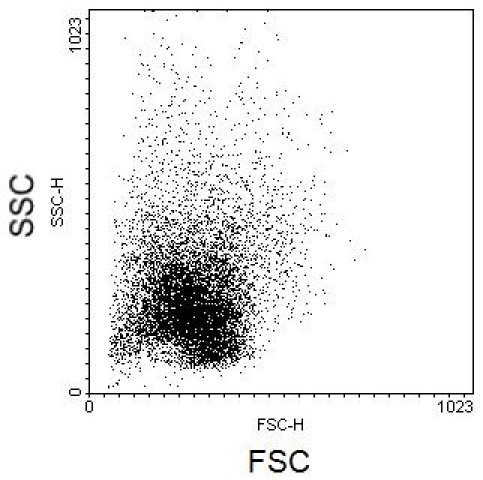
 **Figure 2.** Flow cytometry scatter plot of whole blood sample without erythrocyte lysis with FSC and SSC axes.


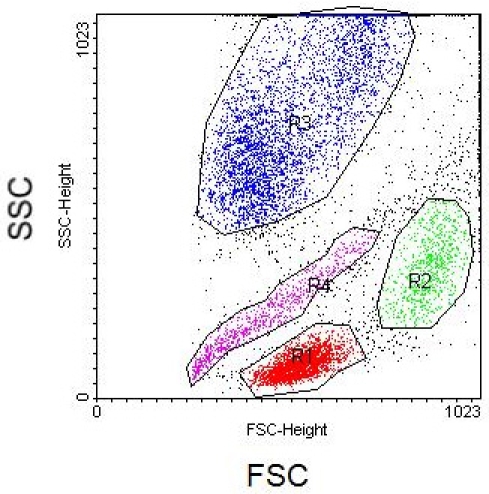
 **Figure 3.** Flow cytometry scatter plot of circulating nucleated cells with microfluidic erythrocyte lysis labeled by populations with FSC and SSC axes. Region 1 (red) represents lymphocytes, Region 2 (green) represents monocytes, Region 3 (blue) represents granulocytes, and Region 4 (purple) has yet to be characterized.

Removal of red cell debris by pipette in the protocol is also important when necessary. Below in Figure 4 is a scatter plot showing a surplus of red cell debris. Although not clouding the scatter plot as in Figure 2, one must account for the added events due to erythrocyte debris during data analysis.


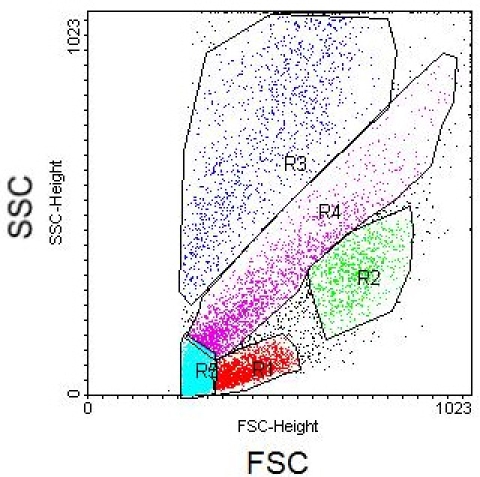
 **Figure 4.** Flow cytometry scatter plot depicting red cell debris, seen as a cluster of events in Region 5 (cyan).

Staining cells for certain antibody phenotype markers will also generate characteristic results. In the following Figures are antibody phenotype stains for 3 distinct leukocyte populations: lymphocytes, monocytes, and granulocytes. Figure 5 depicts lymphocyte populations plotted with axes CD3 and CD4. Monocytes and granulocytes are shown in Figures 6-7 with axes as FSC versus CD14 and CD66b, respectively.


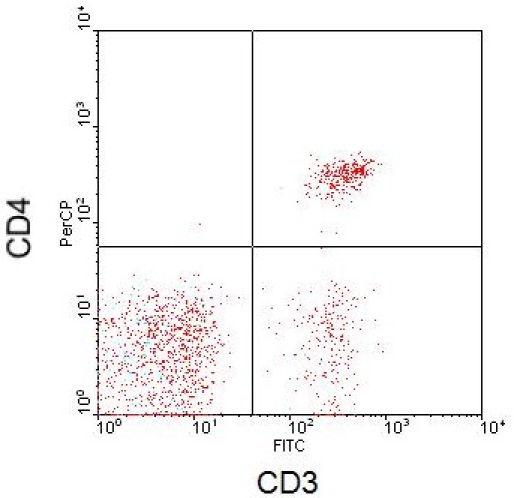
 **Figure 5.** Flow cytometry scatter plot depicting lymphocytes with CD3 and CD4 axes.


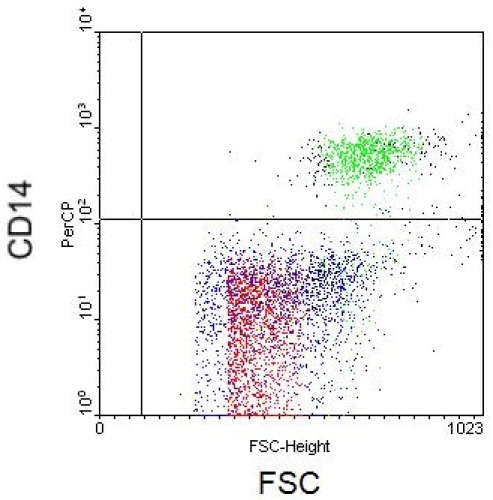
 **Figure 6.** Flow cytometry scatter plot depicting monocytes with FSC and CD14 axes.


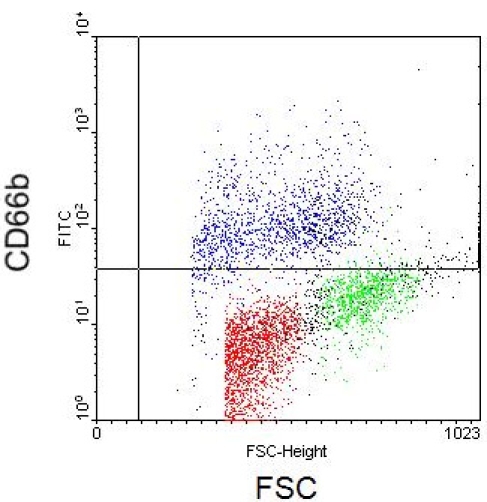
 **Figure 7.** Flow cytometry scatter plot depicting granulocytes with FSC and CD66b axes.

## Discussion

An automated microfluidic blood lysis protocol has been reported. Within the protocol are critical steps worth noting. In section P.2.1, it is important to discard the first blood vial collected as the sample contains dislodged mature endothelial cells acting as false positives. Also make sure to run the blood sample within an hour of collection. Priming the microfluidic device in section P.2.2, it is important to initially rid of the bubbles. Additionally, one should avoid introducing bubbles when attaching new syringes to the needles throughout the protocol. When filling the 1 ml syringe with blood in section P.2.4, one must remember to first add 1X PBS and the blood, all while keeping the syringe vertical to avoid mixture of the two solutions. Before starting the syringe pump pushing the blood sample, ensure the large syringe pump has been running so the system is at full speed. After the sample has finished running through the device and been centrifuged, carefully remove as much supernatant as possible, including red cell debris, without disturbing the white pellet. Lastly, in section P.2.5 follow the manufacturer s protocol to assure the correct concentration of antibody is added to the 100 μL sample.

Applications of the microfluidic protocol are immense in clinical research. Interrogating the immune status of an individual in terms of CNCs generates important information on condition of health and may help with diagnosis and understanding of disease pathogenesis. Accessing these CNC populations through blood sample collection is easier and more convenient than human expression analyses involving tumor tissues. To examine these nucleated cells in circulation one must be able to enrich the populations from other blood components, including erythrocytes, the primary function of the reported device. Hence, such studies would benefit greatly from the microfluidic blood lysis protocol.

Significance of the microfluidic protocol emerges in terms of applications. Because nucleated cell enrichment is required in clinical studies of blood, a protocol requiring little expertise and user-mediated steps that produces clear and consistent results is important. The microfluidic protocol ensures consistency through automation and controlled exposure of cells to harsh environments. With uniformity among clinical laboratories data will be directly comparable [2].
